# Magnetically Recoverable ICT-Functionalized Fe_3_O_4_ Nanoparticles for Efficient Horseradish Peroxidase Immobilization

**DOI:** 10.3390/molecules31010178

**Published:** 2026-01-02

**Authors:** Katarina Isaković, Marko Jonović, Dušan Sredojević, Marko Bošković, Jovana Periša, Zorica Knežević-Jugović, Vesna Lazić

**Affiliations:** 1 Vinča Institute of Nuclear Sciences, National Institute of the Republic of Serbia, Centre of Excellence for Photoconversion, University of Belgrade, 11001 Belgrade, Serbia; katarina.isakovic@vin.bg.ac.rs (K.I.); jburojevic@yahoo.com (J.P.); 2 Institute of Chemistry, Technology and Metallurgy, University of Belgrade, 11000 Belgrade, Serbia; marko.jonovic@ihtm.bg.ac.rs; 3 Laboratory for Theoretical and Condensed Matter Physics, Vinča Institute of Nuclear Sciences, National Institute of the Republic of Serbia, University of Belgrade, 11001 Belgrade, Serbia; markob@vin.bg.ac.rs; 4 Department of Biochemical Engineering and Biotechnology, Faculty of Technology and Metallurgy, University of Belgrade, 11000 Belgrade, Serbia; zknez@tmf.bg.ac.rs

**Keywords:** 5-aminosalicylic acid (5ASA), enzyme immobilization, Fe_3_O_4_ nanoparticles, horseradish peroxidase (HRP), interfacial charge transfer (ICT)

## Abstract

The formation of interfacial charge transfer (ICT) complexes between phenolic ligands and metal oxide surfaces enables surface functionalization strategies with potential applications in catalysis and bioconjugation. In this study, magnetite (Fe_3_O_4_) nanoparticles were modified with two phenolic ligands, 5-aminosalicylic acid (5ASA) and caffeic acid (CA), to generate ICT complexes capable of covalent or non-covalent enzyme immobilization, respectively. The modified nanomaterials were structurally characterized using X-ray diffraction (XRD), transmission electron microscopy (TEM), and Fourier-transform infrared spectroscopy (FTIR). Horseradish peroxidase (HRP) was immobilized on these functionalized supports using varying nanoparticle amounts (10–30 mg) and initial enzyme concentrations (25–250 µg mL^−1^). Catalytic activity was evaluated using pyrogallol oxidation assays. The Fe_3_O_4_/5ASA–HRP system exhibited a maximum activity of 2.5 U per 20 mg of support (approximately 125 U g^−1^), whereas Fe_3_O_4_/CA showed minimal activity under the same conditions. Enzyme loading studies confirmed that 5ASA-enabled covalent attachment resulted in significantly higher immobilization efficiency (up to 1068 mg g^−1^) compared to the CA system. Reusability tests demonstrated that the Fe_3_O_4_/5ASA system retained high absolute catalytic activity during the initial reaction cycles and consistently outperformed the non-covalently immobilized Fe_3_O_4_/CA system upon repeated reuse. The magnetic properties of Fe_3_O_4_ allowed rapid recovery of the biocatalysts using an external magnetic field. These results highlight the effectiveness of ICT-based functionalization for enzyme immobilization, positioning Fe_3_O_4_/5ASA as a promising platform for robust and reusable biocatalysts in environmental and industrial applications.

## 1. Introduction

The development of interfacial charge-transfer (ICT) complexes between phenolic compounds and metal oxide surfaces represents a powerful approach to extending absorption into the visible range, thereby enabling novel photocatalytic and biomedical applications. ICT complexes form through covalent interactions between OH groups from phenolate ligands with OH groups on metal oxide nanoparticle surfaces [1,2,3]. This bonding induces a visible-range red shift in optical absorption, as energy from the phenolic donor is directly transferred to the semiconductor conduction band. In our previous studies, we successfully demonstrated the formation of ICT complexes between bioactive plant extracts and oxide surfaces, such as TiO_2_, ZrO_2_, and CeO_2_, leading to enhanced visible-light properties and biological functions [4,5,6,7,8,9,10,11,12].

Although Fe_3_O_4_ nanoparticles are intrinsically black and do not allow straightforward detection of visible-light absorption shifts via UV/Vis spectroscopy, they possess a hydroxyl-rich surface highly suitable for interfacial charge transfer (ICT) complex formation. Phenolic ligands such as flavonoids or salicylic acid derivatives can form stable ICT complexes by coordinating through their phenolic –OH groups to surface Fe–OH moieties [5]. This interaction not only enhances chemical stability and anchoring of the ligands but also introduces additional functional groups to the nanoparticle interface [1,4,6,13]. For instance, in the case of 5-aminosalicylic acid (5ASA), while the phenolic –OH participates in ICT complex formation, the –NH_2_ group on the opposite end remains accessible and reactive. This spatial orientation enables further surface modification or covalent attachment of biomolecules, such as enzymes. Therefore, ICT complexation with phenolic ligands serves not only as a surface engineering strategy but also as a platform for the multifunctionalization of Fe_3_O_4_ nanoparticles, including enzyme immobilization in biocatalysis and biomedical applications.

Immobilizing enzymes on nanoparticles harnesses advantages such as enhanced stability, easy recovery, reusability, and applicability in complex reaction environments. In addition to the well-established benefits of enzyme immobilization, nanocarriers offer an increased surface area for enzyme binding and enhanced reaction efficiency, further enhancing catalytic performance. While recovery of non-magnetic nanoparticles often requires advanced separation techniques, magnetic Fe_3_O_4_ nanoparticles may facilitate easier separation of immobilized enzymes.

Horseradish peroxidase (HRP), a heme-containing oxidoreductase, has been widely studied in this context and immobilized onto various nanosupports, including polymers, silica, and magnetic nanoparticles, using both physical adsorption and covalent attachment methods [14,15,16,17,18,19,20]. Early works demonstrated the physical adsorption of the enzyme onto unmodified Fe_3_O_4_ nanoparticles, resulting in increased thermal stability and retention of activity post-immobilization, although recovery was limited [15,19,21]. More advanced methods utilize covalent linking via glutaraldehyde or amine-functional silanes on Fe_3_O_4_–SiO_2_ core–shell systems, resulting in enhanced loading, operational stability, and catalytic performance [18,22]. Cavalcante et al. have highlighted the growing importance of magnetically recoverable carriers in enzyme immobilization, emphasizing their operational stability and suitability for industrial applications [23]. Techniques for oriented or tag-mediated immobilization on magnetic nanoparticles add another layer of control and efficiency [24]. Similarly, novel magnetic polymer platforms have demonstrated improved binding and durability in laccase immobilization [25]. In these systems, surface modification is achieved by applying a silica coating, which is subsequently functionalized to introduce –NH_2_ groups that serve as anchoring points for enzyme conjugation.

However, to the best of our knowledge, no study has reported a system where Fe_3_O_4_ nanoparticles are directly functionalized via interfacial charge transfer (ICT) complexation with small, naturally derived organic molecules to create a reactive surface suitable for covalent enzyme immobilization. Despite extensive research on enzyme immobilization on magnetic nanoparticles, most reported systems rely on multistep surface modification strategies, such as silica coating followed by silanization or polymer grafting, which increase synthetic complexity and limit control over surface functionality. In contrast, the direct use of interfacial charge-transfer (ICT) complexes as a surface functionalization tool for enzyme immobilization has remained largely unexplored, particularly for Fe_3_O_4_-based systems. This work addresses this gap by introducing an ICT-based, single-step aqueous functionalization strategy for strong metal oxide–ligand interactions that combines surface stabilization, functional group specificity, and magnetic recoverability.

In the present study, the formation of interfacial charge-transfer (ICT) complexes is primarily employed as a surface functionalization strategy rather than for photocatalytic activation. ICT complexation anchors phenolic ligands to the Fe_3_O_4_ surface, introducing well-defined, spatially accessible functional groups that facilitate enzyme attachment and covalent bond formation. Horseradish peroxidase (HRP) was selected as a model enzyme to evaluate the effectiveness of this ICT-based surface functionalization in promoting enzyme immobilization, retaining activity, and improving operational stability. HRP is a well-established redox enzyme whose catalytic performance is susceptible to immobilization chemistry and surface microenvironment. Moreover, HRP is widely employed in the degradation of organic pollutants, which aligns with the long-term objective of extending this ICT-functionalized system toward oxidative degradation of organic contaminants, to be addressed in future studies.

Within this framework, enzyme immobilization was achieved through two fundamentally different mechanisms enabled by the chosen ligands. Functionalization with 5-aminosalicylic acid allows covalent attachment of HRP via the free amino group, resulting in strong enzyme anchoring and improved resistance to desorption. In contrast, caffeic acid–modified Fe_3_O_4_ provides a surface that promotes enzyme immobilization predominantly through physical adsorption, involving hydrogen bonding and electrostatic interactions. The use of these two ligand systems, therefore, enables a direct comparison between covalent and non-covalent immobilization strategies on structurally identical magnetic supports.

In this work, we explore a novel approach that integrates ICT-complex design with enzyme immobilization by functionalizing Fe_3_O_4_ nanoparticles with 5-aminosalicylic acid (5ASA) and caffeic acid (CA), enabling covalent and non-covalent binding of the enzyme. Although the intrinsic black color of Fe_3_O_4_ could hinder direct monitoring of ICT-related optical changes, the successful surface modification was confirmed through structural characterization, including X-ray diffraction (XRD) and transmission electron microscopy (TEM) for nanoparticle morphology, and Fourier-transform infrared spectroscopy (FTIR) for analyzing the functional groups before and after enzyme immobilization. The immobilization efficiency of horseradish peroxidase (HRP) will be evaluated using both ligands, and enzymatic activity will be systematically studied by varying the enzyme-to-nanoparticle ratio and the initial enzyme concentration. Reusability tests will be assessed through multiple catalytic cycles. The aim is to develop magnetically retrievable and catalytically efficient nanobiocatalysts, with potential applications in biosensing, green catalysis, and wastewater treatment.

## 2. Results and Discussion

### 2.1. Synthesis and Characterization of Fe_3_O_4_ Nanoparticles

**Figure 1:**
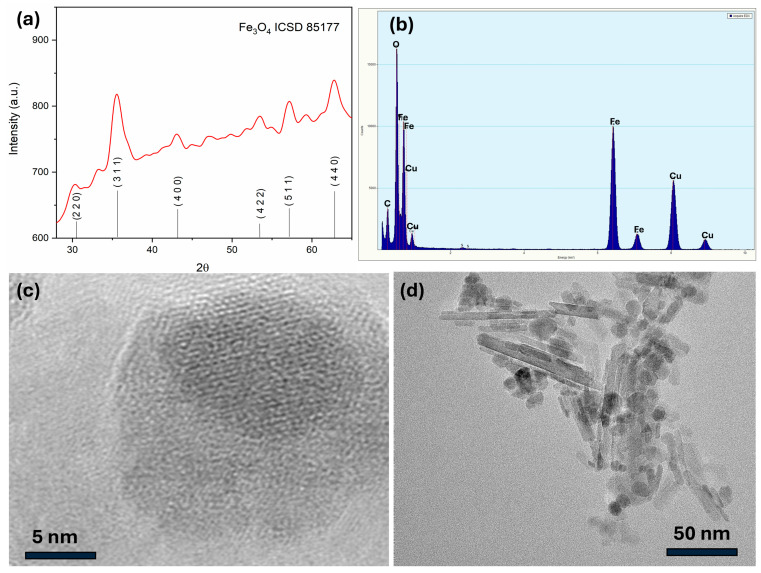
Characterization of Fe_3_O_4_ nanoparticles: (**a**) XRD pattern, (**b**) EDX spectrum, (**c**,**d**) high-magnification TEM images.

The wide-angle XRD pattern of synthesized Fe_3_O_4_ nanoparticles is presented in Figure 1a. The diffractogram displayed the presence of several distinct peaks at 30.2, 35.6, 43.2, 53.7, 57.3, and 62.9° that belong to (220), (311), (400), (422), (511), and (440) planes of the inverse spinel crystalline Fe_3_O_4_, respectively (JCPDS Card No.85177).

Transmission electron microscopy (TEM) was used to perform a detailed morphological analysis of the Fe_3_O_4_ nanoparticles. As shown in the low-magnification TEM images (Figure 1c,d), the sample contained predominantly spherical nanoparticles with diameters of approximately 10 nm and rod-like structures measuring roughly 100 nm in length and 5 nm in width. The coexistence of spherical and rod-shaped nanoparticles is likely a consequence of the different iron salts, surfactants, or solvents used during the synthesis process [26,27]. Elemental analysis performed using energy-dispersive X-ray spectroscopy (EDX) (Figure 1b) confirmed the presence of iron as the primary component, along with oxygen and carbon, following the composition of the synthesized nanocomposite. Copper signals detected in the EDX spectrum originated from the copper grid used for sample mounting.

The textural properties of the Fe_3_O_4_ nanoparticles were evaluated by nitrogen adsorption–desorption (BET) analysis. The nanoparticles exhibited a specific surface area of 117 m^2^ g^−1^ and an average pore diameter of approximately 16 nm. These characteristics provide sufficient accessible surface area and pore dimensions for enzyme immobilization, allowing adequate accommodation of HRP molecules without severe steric constraints.

Although Fe_3_O_4_ nanoparticles are often reported with near-spherical morphology, the presence of both particle-like and rod-like structures in the present system introduces additional structural advantages. Anisotropic (rod-like) morphologies provide increased surface area and expose different crystallographic facets, thereby enhancing the density and accessibility of surface hydroxyl groups. This structural diversity facilitates more effective anchoring of phenolic ligands (5-ASA and CA) and improves the spatial accommodation of HRP molecules, thereby promoting higher enzyme loading and activity.

### 2.2. Immobilization of HRP to Fe_3_O_4_/5ASA and Fe_3_O_4_/CA Nanocomposite Materials

**Scheme 1:**
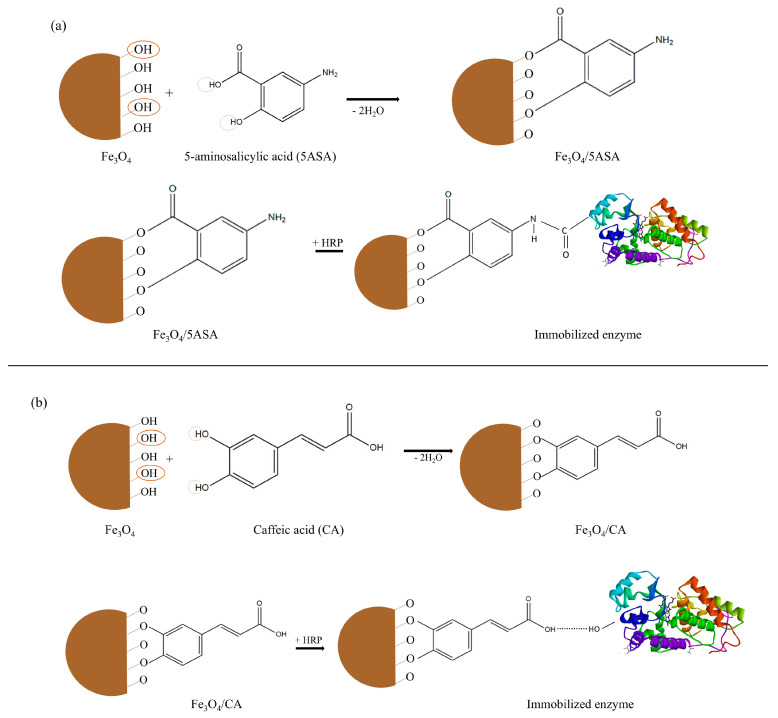
Schematic illustration of the immobilization of horseradish peroxidase (HRP) onto Fe_3_O_4_ nanoparticles functionalized with (**a**) 5-aminosalicylic acid (5ASA) via interfacial charge transfer (ICT) complex formation and covalent binding through the amino group of 5ASA, (**b**) caffeic acid (CA) via interfacial charge transfer (ICT) complex formation and hydrogen binding with CA.

Scheme 1 illustrates the immobilization strategies employed in this study, highlighting the fundamental differences between covalent attachment of HRP via 5-aminosalicylic acid (5ASA) and non-covalent adsorption mediated by caffeic acid (CA). The activation of carboxyl groups using EDAC and subsequent amide bond formation between HRP and surface-exposed amino groups of 5ASA are explicitly shown.

**Scheme 2:**
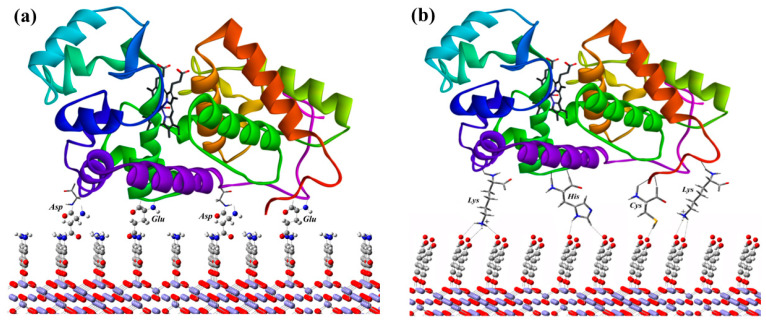
Enzyme peroxidase is pictorially represented as immobilizing on the surface of Fe_3_O_4_ nanoparticles modified with (**a**) 5ASA and (**b**) CA. The amide bonds between the amino groups of 5ASA and the amino acid residues of *Glu* and *Asp* are indicated. The interactions between the COO groups of CA and the amino acid residues of *Lys*, *His*, and *Cys* are denoted with dotted lines. (gray: carbon; white: hydrogen; red: oxygen; blue: nitrogen; yellow; sulfur; purple: iron).

At the operational pH of 8.5, the enzyme peroxidase is immobilized onto the surface of Fe_3_O_4_ nanoparticles modified with 5ASA. Since the amino group of 5ASA has a pKa value of 5.8, it is deprotonated at this pH, which favors nucleophilic attack on carboxylate groups of Glu and Asp residues in the enzyme. This suggests that the enzyme is covalently bonded to the Fe_3_O_4_/5ASA surface via the formation of amide bonds between the –NH_2_ groups of 5ASA and the carboxylate groups of *Glu* and *Asp* residues (Scheme 2a). The catalytic concentration of ferric and ferrous ions in the buffer solution may promote the formation of the amide bond [1]. On the other hand, the peroxidase enzyme cannot be immobilized onto the Fe_3_O_4_ NPs modified by caffeic acid (CA), likely due to the absence of covalent interactions. Instead, the enzyme is assumed to bind non-covalently to the surface -COO^–^ groups of CA through hydrogen bonds and salt bridges (Scheme 2b). Hydrogen bonding can occur through amino acid residues such as Arg, His, Lys, and Cys, among others. At the same time, salt bridges may be formed between CA and *Arg*, *Lys*, and *Pro* residues that are protonated in the buffer solution (pH = 8.5). This could explain why the enzymes cannot form covalent bonds with Fe_3_O_4_/CA NPs. Since these residues have high pKa values (12.5, 10.8, and 10.6, respectively), they remain protonated at this pH, which prevents nucleophilic attack and thus the formation of amide bonds with CA.

**Figure 2:**
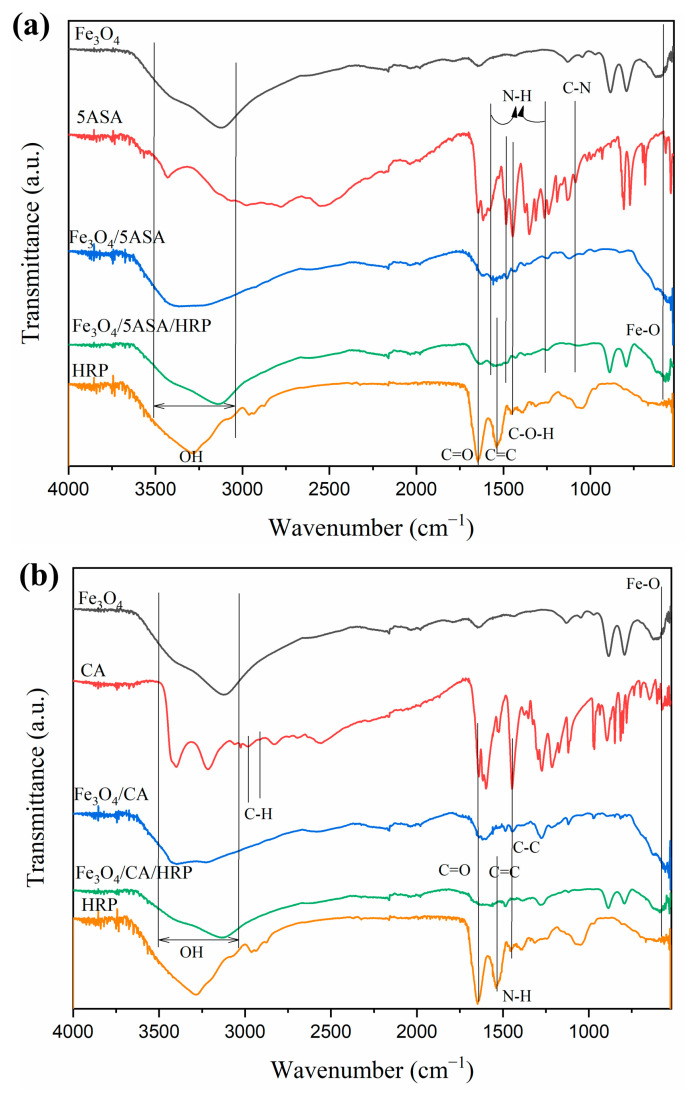
FTIR spectra of Fe_3_O_4_ nanoparticles and composite nanomaterials (**a**) Fe_3_O_4_/5ASA and (**b**) Fe_3_O_4_/CA before and after immobilization with HRP.

FTIR analysis was conducted to better characterize the interactions between HRP and functionalized Fe_3_O_4_ nanoparticles, and the results are presented in Figure 2a Fe_3_O_4_/5ASA and Figure 2b Fe_3_O_4_/CA). The absorption peak at 580 cm^−1^ was the vibration of the Fe–O bond [28,29].

Figure 2 shows the FTIR spectra of the Fe_3_O_4_ nanoparticles, free ligands (CA and 5ASA), the enzyme HRP, and corresponding hybrid materials before and after immobilization. The absorption peak at 560 cm^−1^ corresponded to the Fe–O bond vibration [28]. The peaks between 3000 and 3500 cm^−1^ are due to the O-H stretching vibration from hydroxyl groups on nanomaterials [29]. The FTIR absorption spectrum of the 5ASA is shown in Figure 2a. Notably, a band of average intensity at 1647 cm^−1^ corresponds to the asymmetric stretch of the C=O bond and bands of intensity at 1446 cm^−1^ and 1485 cm^−1^ corresponding to the angular and axial deformations of the C–O–H and stretches of the aromatic ring C=C bonds, respectively, confirming the presence of a carboxylic group in the chemical structure of the 5ASA [30]. The bands at 1574 and 1263 cm^−1^, assigned to N–H vibrations, almost disappear after enzyme immobilization, suggesting that the amino group participates in amide bond formation with the enzyme. As shown in Figure 2, peaks observed at 1637, 1520, and 1440 cm^−1^ can be assigned to the amide I band, amide II band, and NH-related vibrational modes (amide III), respectively, confirming the presence of immobilized HRP on the nanoparticle surface [31]. The FTIR absorption spectrum of the CA is shown in Figure 2b. Bands at 2982 and 2912 cm^−1^ are ascribed to the C-H vibration modes [32]. The nanomaterial modified with caffeic acid exhibits a carbonyl-associated peak in all three samples, both before and after enzyme immobilization, suggesting that no covalent bonding occurred between the enzyme and the material. The intense band at 1645 cm^−1^ is assigned to the stretching of the carbonyl group (C=O) from the CA. Furthermore, the intense bands at 1450 cm^−1^ are attributed to olefinic C-C stretching modes.

**Figure 3:**
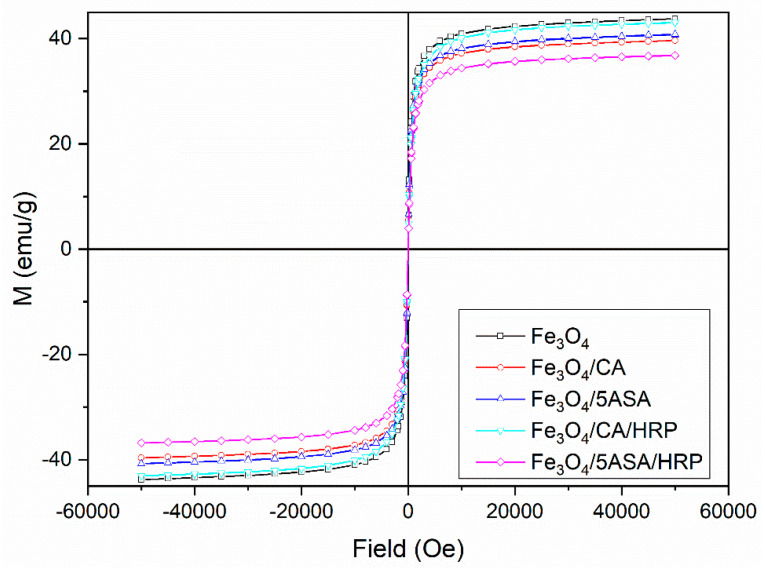
Magnetization curves of Fe_3_O_4_, Fe_3_O_4_/CA, and Fe_3_O_4_/5ASA before and after immobilization with HRP.

The magnetization curves of Fe_3_O_4_, Fe_3_O_4_/CA, and Fe_3_O_4_/5ASA before and after HRP enzyme immobilization were measured at 300 K in a magnetic field range of −50 to +50 kOe, as shown in Figure 3. The saturation magnetization of pristine Fe_3_O_4_ was 45 emu/g. After functionalization with caffeic acid and 5-aminosalicylic acid, the saturation magnetization values slightly decreased to 40 emu/g (Fe_3_O_4_/CA) and 41 emu/g (Fe_3_O_4_/5ASA), which can be attributed to the presence of a non-magnetic organic phase on the surface of the magnetic nanoparticles. Upon enzyme immobilization, a further reduction in magnetization was observed for the Fe_3_O_4_/5ASA sample (37 emu/g), consistent with the increased proportion of non-magnetic material resulting from the enzyme’s presence. In contrast, the slight increase in Ms (40 to 43 emu g^−1^) observed after HRP immobilization on Fe_3_O_4_/CA is slight. It may reflect minor variations in organic surface coverage and/or sample-to-sample heterogeneity rather than a systematic magnetic enhancement. Since CA-based immobilization relies predominantly on non-covalent interactions under alkaline conditions (pH 8.5), partial rearrangement or reduction in the organic layer during the immobilization/washing steps cannot be excluded. These variations in magnetization values reflect the relative contributions of magnetic and non-magnetic components within the composite mass and are consistent with expectations for organic-inorganic hybrid nanostructures [20].

### 2.3. Activity of HRP Immobilized to Fe_3_O_4_/5ASA and Fe_3_O_4_/CA Nanocomposite Materials

**Figure 4:**
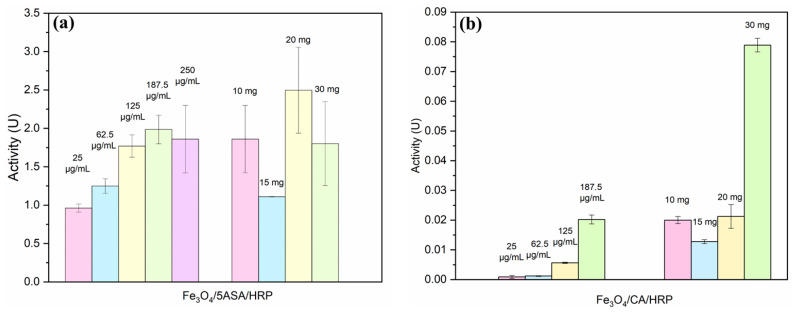
Enzyme activity (U) of HRP immobilized on (**a**) Fe_3_O_4_/5ASA and (**b**) Fe_3_O_4_/CA. Left bars: varying enzyme concentrations (25–250 µg mL^−1^) with 10 mg of nanoparticles. Right bars: varying nanoparticle amounts (10–30 mg) with 250 µg mL^−1^ of enzyme.

Enzyme activity is expressed in units (U), where one unit (1 U) corresponds to the amount of HRP that catalyzes the oxidation of 1 µmol of pyrogallol to purpurogallin per minute under the assay conditions described in Section 3.5. The results presented in Figure 4 clearly show that HRP immobilized on Fe_3_O_4_/5ASA nanomaterials exhibited significantly higher catalytic activity than that on Fe_3_O_4_/CA under all tested conditions. For the condition with 10 mg of nanoparticles (Figure 4a, left set), activity increased with enzyme concentration, reaching its highest value at 187.5 µg mL^−1^. Activity increases slightly at a higher enzyme concentration (250 µg mL^−1^). This effect is consistent with the non-monotonic loading-activity relationship often observed for immobilized enzymes: excessive surface coverage can cause steric crowding and shielding of active sites, increase diffusional limitations, and promote partial inactivation upon exposure to H_2_O_2_ [33]. Even with this decrease at 250 µg mL^−1^, the highest measured value for Fe_3_O_4_/5ASA (2.5 U with 20 mg of nanoparticles) corresponds to over 125 U g^−1^ of nanomaterial, indicating excellent catalytic performance after immobilization. This superior activity can be attributed to the covalent bonding between HRP and the 5-aminosalicylic acid-modified surface, which likely creates a stable, favorable microenvironment for the enzyme, thereby limiting conformational changes and preventing leaching during washing and reuse. In contrast, caffeic acid (CA)-functionalized nanoparticles enable only non-covalent interactions (e.g., hydrogen bonding, ionic bridges), which are less stable under operational conditions, as reflected by both the low initial activity (max 0.08 U) and the rapid loss in performance upon repeated use. The low activity of the Fe_3_O_4_/CA system can be attributed to the molecular orientation of caffeic acid on the Fe_3_O_4_ surface, where –COOH groups remain exposed and do not participate in covalent coupling with the enzyme. As a result, HRP is bound predominantly through weak non-covalent interactions such as hydrogen bonding and ionic bridges, leading to reduced catalytic stability under operational conditions. Immobilization yields were determined by measuring the total protein concentration in the immobilization supernatant using the Bradford assay. This approach is commonly employed for HRP immobilization studies and allows reliable comparison of enzyme loading under identical experimental conditions. Since a commercially purified HRP preparation was used, the measured protein content is dominated by HRP.

Data from Table S1 further support these findings: for Fe_3_O_4_/5ASA, the amount of immobilized enzyme reached up to 1068.7 mg g^−1^ of support, with 21.4 mg of HRP retained on 20 mg of nanoparticles, while a substantial portion of the enzyme remained in the reaction mixture when CA-functionalized materials were used, typically under 5 mg g^−1^, with most values falling below the detection limit. For these samples, enzyme loading was at or below the Bradford assay detection limit, and in some cases could not be quantified with confidence (reported as ND in Table S1). This pronounced difference demonstrates that 5ASA-functionalization not only promotes covalent binding but also facilitates substantially higher enzyme loading, directly contributing to enhanced catalytic performance. As discussed by Boudrant et al. [34], the evaluation of immobilization efficiency can be based on protein balance or enzyme activity balance, depending on the enzyme system and experimental constraints. In the case of HRP, activity-based quantification in the supernatant can be affected by oxidative inactivation and assay interference, particularly in the presence of hydrogen peroxide. Therefore, in the present study, protein-based immobilization yields were combined with direct assessment of the expressed activity and reusability of the immobilized enzyme, which represent the most relevant indicators of functional enzyme–support interactions.

Although literature values for HRP activity expressed in U g^−1^ of support are scarce, similar systems employing covalent immobilization strategies, such as HRP on modified silica or synthetic polymer beads, report activity in the range of 200–800 U g^−1^ [35,36], depending on enzyme loading and surface properties. Therefore, the achieved values in this study are comparable or superior to many existing immobilization systems, particularly considering the simplicity of the Fe_3_O_4_-based platform and the mild immobilization conditions employed. These results highlight the importance of rational surface functionalization and immobilization chemistry in designing nanomaterials that retain or even enhance the catalytic activity of enzymes after attachment.

Numerous approaches for horseradish peroxidase immobilization have been reported, including physical adsorption on magnetic nanoparticles, covalent attachment via silica-amine-glutaraldehyde linkers, and immobilization on polymeric or ceramic supports. While physical adsorption on bare Fe_3_O_4_ nanoparticles offers simplicity, such systems often suffer from enzyme leaching and rapid loss of activity during repeated use [15,19,21]. Covalent immobilization strategies based on silica-coated magnetic nanoparticles and silane coupling agents generally provide improved stability and higher enzyme loading; however, they require multistep surface modification procedures, the use of organic solvents, and additional functionalization steps to introduce reactive groups [18,22,23]. Polymer-based and ceramic supports can further enhance stability, but often require complex synthesis routes or reduced mass-transfer efficiency [31,35,36]. In comparison, the ICT-based functionalization strategy employed in this work enables direct surface modification of Fe_3_O_4_ nanoparticles using small, naturally derived phenolic ligands, eliminating the need for silica coating or silanization. The use of 5-aminosalicylic acid provides a well-defined reactive amino group for covalent enzyme attachment while preserving mild, aqueous immobilization conditions. As a result, the Fe_3_O_4_/5ASA system exhibits high enzyme loading, competitive catalytic activity (>100 U g^−1^), and superior operational stability compared to non-covalent systems and many previously reported magnetic supports. Notably, the enhanced activity of the covalently immobilized HRP cannot be attributed solely to increased enzyme loading. The ICT-mediated anchoring of 5ASA provides a spatially favorable immobilization geometry, in which covalent attachment occurs through a flexible, surface-exposed amino group, minimizing steric constraints and preserving active-site accessibility. This combination of high loading capacity and maintained enzyme conformation distinguishes the present system from conventional covalent immobilization approaches, where activity losses are frequently observed.

Nevertheless, certain limitations of the present approach should be acknowledged. Partial activity loss during repeated cycles is observed, which may arise from oxidative deactivation of HRP by hydrogen peroxide, gradual conformational changes in the immobilized enzyme, or diffusional limitations associated with surface crowding. Additionally, while magnetic separation is highly advantageous at the laboratory scale, further optimization will be required to address nanoparticle aggregation and enzyme distribution in larger-scale systems. Overall, the ICT-assisted functionalization strategy strikes a balance among simplicity, stability, and performance, positioning Fe_3_O_4_/5ASA nanoparticles as a competitive alternative to conventional multistep immobilization platforms.

**Figure 5:**
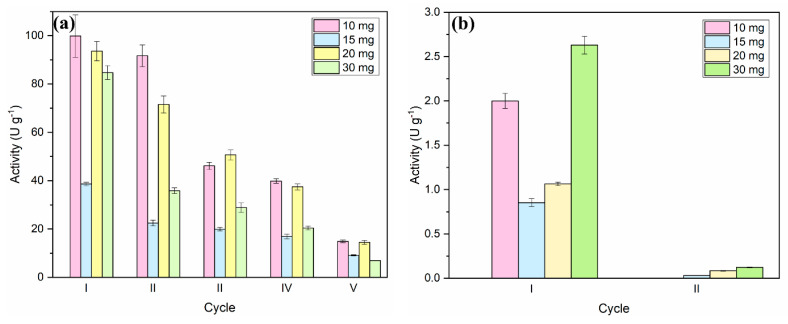
Activity in cycles of peroxidase immobilized on (**a**) Fe_3_O_4_/5ASA nanoparticles and (**b**) Fe_3_O_4_/CA nanoparticles vs. different amounts of nanoparticles used during the immobilization, with 250 µg mL^−1^ of enzyme.

The results shown in Figure 5 demonstrate that HRP immobilized on Fe_3_O_4_/5ASA nanomaterials exhibits only a moderate decrease in activity after the first two catalytic cycles (approximately 10% loss for 10 mg, 20% loss for 15 mg, 25% loss for 20 mg of nanoparticles). For 30 mg of nanoparticles, the activity decreases by about 50% after the second cycle. The sharp initial decrease, pronounced after the first cycle, likely results from the removal of loosely adsorbed or weakly bound enzyme molecules during washing and reaction. At the same time, the subsequently retained activity reflects the fraction of the enzyme covalently attached to the support. Afterward, a more gradual decline of up to ~80% is observed from the third cycle onward, indicating progressive inactivation of the firmly bound enzyme fraction. This loss is attributed to partial enzyme inactivation resulting from repeated exposure to hydrogen peroxide and pyrogallol, which leads to oxidative damage of the heme group and nearby amino acids, as well as potential conformational changes induced by the reaction conditions. Additionally, gradual blocking of active sites by oxidation products may further contribute to activity loss. The lower activity observed at an intermediate nanoparticle loading (15 mg) suggests that a delicate balance among enzyme surface density, nanocarrier dispersion, and substrate diffusion governs catalytic performance. Such non-monotonic behavior is characteristic of immobilized enzyme systems, in which optimal activity is achieved within a specific window of enzyme-to-support ratio.

Nevertheless, the residual activity obtained after five cycles remains higher than that of the Fe_3_O_4_/CA system and several non-covalent immobilization platforms reported in the literature [31,37], confirming the competitive stability of the Fe_3_O_4_/5ASA nanobiocatalysts. This high level of operational stability was particularly observed for nanobiocatalysts prepared using higher amounts of nanoparticles (20–30 mg) during the immobilization step, i.e., at a lower enzyme-to-nanocarrier ratio, indicating that improved enzyme distribution and reduced surface crowding contribute to better performance after the initial washout of weakly bound enzyme. The enhanced performance is attributed to covalent bonding between the enzyme and the amino-functionalized nanoparticle surface, which likely stabilizes the enzyme’s structure and prevents leaching. In addition to the high catalytic activity and stability observed, the Fe_3_O_4_/5ASA system offers several practical advantages over many reported immobilization platforms, including the formation of a stable ICT complex with covalent metal oxide-ligand binding, a simple and reproducible functionalization procedure performed entirely in aqueous medium without generating unnecessary waste, and the use of biocompatible ligands, making the process environmentally friendly and suitable for potential biomedical applications. In contrast, HRP immobilized on Fe_3_O_4_/CA, where only non-covalent interactions are present, shows a rapid decline in activity, losing approximately 90% of its initial value after only two cycles. When explicitly compared with other magnetic nanoparticle-based immobilization systems reported in the literature, these results confirm that covalent immobilization via 5-aminosalicylic acid provides not only high initial activity (as shown in Figure 4) but also maintains a competitive absolute activity level after multiple reuses, making this system highly promising for biotechnological applications requiring robust and reusable biocatalysts.

From a practical perspective, the proposed functionalization and immobilization strategy is well-suited to scaling up, as it is based on a single-step aqueous process, uses inexpensive and biocompatible ligands, and avoids organic solvents or specialized equipment. The magnetic nature of the carriers enables rapid and straightforward separation from the reaction media using an external magnetic field, which is particularly advantageous in both continuous and batch processes. As shown in Figure 5, the Fe_3_O_4_/5ASA nanobiocatalysts retain a high absolute activity even after five reuse cycles, despite a decline over time, suggesting that the system can maintain satisfactory performance in extended operations. Potential limitations for industrial application include partial activity loss after multiple cycles, the need to optimize nanoparticle dispersion to minimize agglomeration in larger volumes, and the cost of enzyme replenishment. These aspects can be addressed in future studies through process optimization, improved immobilization chemistries, or enzyme engineering, paving the way for pilot-scale and industrial implementation.

**Figure 6:**
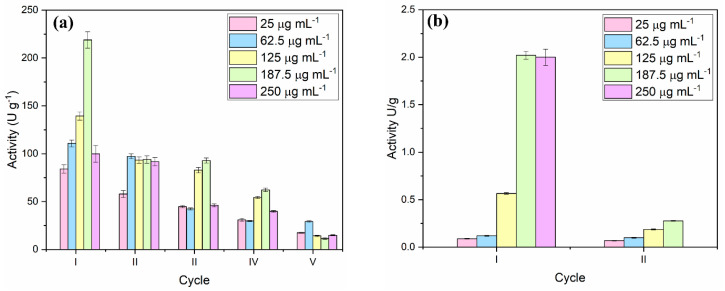
Activity in cycles of peroxidase immobilized on (**a**) Fe_3_O_4_/5ASA nanoparticles and (**b**) Fe_3_O_4_/CA nanoparticles vs. different concentrations of enzyme, with 10 mg of material.

Figure 6 illustrates how varying the initial enzyme concentration affects the catalytic durability of HRP immobilized on Fe_3_O_4_/5ASA and Fe_3_O_4_/CA supports over five reuse cycles. In the Fe_3_O_4_/5ASA system, all tested concentrations maintained relatively high activity during the first two to three cycles, indicating that the enzyme molecules covalently attached to the 5ASA-functionalized surface remain structurally stable under reaction conditions. A significant decrease in activity was observed at an enzyme concentration of 187.5 µg mL^−1^, with a drop of about 85% after five cycles. In contrast, samples prepared with moderate enzyme loadings (62.5–125 µg mL^−1^) retained 40–50% of their activity even after four cycles, suggesting that lower initial enzyme concentrations provide better long-term stability. This trend likely results from reduced steric crowding and more uniform surface distribution of the enzyme, which minimizes conformational stress and facilitates substrate diffusion. Conversely, higher enzyme concentrations yield the highest initial activity but also greater susceptibility to inactivation during repeated use. The Fe_3_O_4_/CA system, on the other hand, exhibited a much faster decline in activity across all tested concentrations, losing most of its catalytic efficiency after only two cycles. This behavior reflects the weaker, non-covalent nature of enzyme binding in CA-functionalized materials and confirms the superior stabilization achieved via covalent immobilization through 5ASA.

When compared with relevant literature, the Fe_3_O_4_/5ASA system demonstrates competitive performance. HRP immobilized on mesoporous magnetic silica supports retained about 70% of its activity after ten cycles [31], while ceramic-based composites maintained around 66% after ten uses [38]. Moreover, an HRP-mesoporous composite exhibited just 50% activity after five cycles, illustrating significant enzyme loss [39]. In our system, although a gradual loss of activity occurs with repeated use, Fe_3_O_4_/5ASA–HRP prepared with moderate enzyme concentrations (62.5–125 µg mL^−1^) retains 40–50% of its activity even after four cycles, which is comparable to or higher than that of many reported magnetic nanocarriers under similar conditions. This stability, combined with the simplicity of the functionalization procedure and the biocompatible nature of 5ASA, highlights the potential of this system for applications where reusability and environmental safety are critical, even if partial enzyme deactivation occurs over multiple cycles.

The excellent catalytic performance of Fe_3_O_4_/5ASA/HRP was further supported by reusability experiments (Figure 5 and Figure 6), which demonstrated that both the amount of nanomaterial and the initial enzyme concentration play crucial roles in operational stability. Increasing the quantity of nanoparticles (Figure 5) led to a slower decline in activity, with the highest stability observed at 20–30 mg, where activity loss after five cycles was notably lower than at smaller nanoparticle amounts. Varying the initial enzyme concentration (Figure 6) revealed that, although all Fe_3_O_4_/5ASA samples retained high activity in the first few cycles, the sample with the loading of 187.5 µg mL^−1^ experienced a nearly 85% decrease after five consecutive cycles. In contrast, lower initial enzyme concentrations resulted in minor losses after four cycles, suggesting that lower loading may be advantageous when extended reuse is required. In contrast, higher loading is beneficial when maximal initial activity is prioritized for short-term applications. This behavior likely reflects differences in enzyme packing density and active-site accessibility, as well as the effects of oxidative stress and product accumulation during repeated use. In contrast, Fe_3_O_4_/CA-based systems exhibited a sharp decline in activity across all conditions, indicating that non-covalent binding is insufficient to provide stability under operational stresses. Together, these findings confirm that covalent immobilization via 5ASA not only enables high catalytic activity but also provides a tunable strategy for balancing immediate performance and long-term operational stability, critical parameters for practical biocatalytic applications.

As shown in Figure 4, Figure 5 and Figure 6, the key outcome of the activity assays is the consistently higher activity and improved reusability of the Fe_3_O_4_/5ASA system compared to Fe_3_O_4_/CA across all tested conditions, despite variations in enzyme concentration and nanoparticle amount. Although individual activity values vary with experimental conditions, the figures collectively demonstrate that covalent immobilization via 5ASA provides a clear advantage in terms of both initial activity and operational stability.

Numerous HRP immobilization strategies have been reported using a wide range of carrier materials, including polymeric beads, silica-coated magnetic nanoparticles, carbon-based supports, and hybrid inorganic–organic matrices. While many of these systems achieve acceptable enzyme loading, they often involve multistep surface modification procedures or rely on purely adsorptive interactions, which may limit reusability. For example, silica-based magnetic supports functionalized with silane linkers typically exhibit HRP loadings of 10–40 mg g^−1^, with specific activities below 100 U g^−1^. In contrast, polymeric or sol–gel matrices often suffer from diffusional limitations despite improved stability. In contrast, the ICT-functionalized Fe_3_O_4_/5ASA system presented in this work combines a simple surface functionalization route with covalent enzyme attachment, resulting in competitive enzyme loading (>1000 mg g^−1^) and high specific activity (>200 U g^−1^). Importantly, compared to previously reported magnetic systems, the present approach avoids silica coating and complex coupling chemistries while maintaining efficient magnetic recovery and operational stability. These features position the Fe_3_O_4_/5ASA platform as a promising and practically attractive alternative for HRP immobilization.

## 3. Materials and Methods

Horseradish peroxidase (HRP, EC 1.11.1.7) was obtained from Sigma-Aldrich (St. Louis, MO, USA) as a commercially purified enzyme preparation with a reported specific activity of approximately 250 U mg^−1^ (purpurogallin units). HRP is a monomeric heme-containing glycoprotein with a molecular mass of about 44 kDa, a hydrodynamic diameter of roughly 5–6 nm, and an isoelectric point (pI) in the range of 7.2–9.0, depending on the isoform. According to the manufacturer’s specifications, the enzyme purity exceeds 90%, as confirmed by internal quality control. As with most commercially purified plant-derived enzymes, minor amounts of non-enzymatic proteins may be present; however, all immobilization experiments were performed using the same enzyme batch, ensuring consistent comparative evaluation of immobilization efficiency and catalytic performance.

Hydrochloric acid 37% was obtained from Acros Organic (Newark, NJ, USA). Iron (III) chloride hexahydrate was obtained from Carlo Erba Reagents S.A.S. (Val-de-Reuil, France). Hydrogen peroxide (H_2_O_2_) 3% (0.97 M) was purchased from Galafarm (Belgrade, Serbia). Sodium dihydrogen phosphate, disodium hydrogen phosphate, and acetic acid were obtained from Centrohem (Stara Pazova, Serbia). 1-ethyl-3-(3-dimethylaminopropyl) carbodiimide (EDAC) was obtained from Thermo Fisher Scientific (Waltham, MA, USA). Ammonium hydroxide was obtained from NPK Engineering (Belgrade, Serbia). 5-aminosalicylic acid and pyrogallol were obtained from Alfa Aesar (Karlsruhe, Germany), while caffeic acid was obtained from TCI Development Co. (Shanghai, China). Other chemicals used in this work were of commercial analytical grade. Milli-Q deionized water was used as the solvent with a resistivity of 18.2 MΩ cm^−1^.

### 3.1. Synthesis of Magnetic Nanoparticles

Fe_3_O_4_ nanoparticles were synthesized using a co-precipitation method in which ammonium hydroxide (NH_4_OH) served as the precipitating agent [5]. In this procedure, FeCl_3_ and FeSO_4_ were co-precipitated with a 10% excess of Fe^2+^ to compensate for partial oxidation of Fe^2+^ to Fe^3+^ during synthesis, which can occur in the presence of dissolved oxygen. This excess ensures that the required stoichiometric Fe^2+^:Fe^3+^ ratio of 1:2 is maintained throughout the reaction, allowing for the formation of phase-pure magnetite with optimal magnetic properties. For every experiment, 13.5 g of FeCl_3_·6H_2_O and 7.65 g of FeSO_4_·7H_2_O were dissolved in 20 mL of distilled water. The precipitation was carried out at room temperature (22–24 °C) under continuous vigorous magnetic stirring (~800 rpm). Concentrated ammonium hydroxide (25% NH_4_OH, 17 mL) was added dropwise to the iron salt solution, with continuous monitoring of the pH using a calibrated pH meter to maintain a pH of approximately 11 during precipitation. The resulting mixture was then diluted with distilled water to a total volume of 150 mL, stirred for approximately 30 min, and then filtered. The solid product was separated from the reaction mixture using a strong magnetic field and washed repeatedly with 1000 mL of distilled water (over 7–8 cycles) until the pH stabilized at around 7. Subsequently, the washed magnetite powder was redispersed in 100 mL of distilled water, and the pH was adjusted to 2 to promote peptization. The colloidal dispersion was dialyzed against 1 mM HCl until the pH settled to approximately 3, then stored overnight at 4 °C. Finally, the colloidal magnetite was transferred into a volumetric flask and stored in the dark at 4 °C for further use. Afterward, the dispersion was dried in an oven at 45 °C before characterization and functionalization. Representative photographs of the synthesis steps are shown in Figure S1a–d (Supplementary Information).

### 3.2. Preparation of Fe_3_O_4_ with 5-Aminosalicylic and Caffeic Acid

The appropriate ligands (89 mg of 5ASA and 104.8 mg of CA) were dissolved under continuous stirring using a magnetic stirrer with gentle heating (50 mL of distilled water per 100 mg of Fe_3_O_4_). After the complete dissolution of the ligands, magnetic nanoparticles were introduced into the solution (in a molar ratio of Fe_3_O_4_ to ligands of 1:1.35) and incubated at room temperature in the dark for three days. The ligand-to-Fe_3_O_4_ molar ratio of 1:1.35 was selected based on the estimated density of surface –OH groups (~10 groups per nm^2^) calculated from the specific surface area of the nanoparticles, as determined in our previous studies [5]. This estimation is based on the widely accepted surface chemistry of magnetite nanoparticles, which exhibit a hydroxyl-rich surface under aqueous conditions. The assumed –OH group density (~10 groups nm^−2^), derived from the specific surface area and literature-reported values for Fe_3_O_4_, provides a reasonable approximation of the number of available anchoring sites for phenolic ligands forming ICT complexes. This amount provides sufficient ligand to achieve complete surface coverage without introducing excess unbound ligand. After incubation, the dispersion was transferred to a cuvette and thoroughly mixed with deionized water using a vortex mixer to facilitate washing and remove any unbound ligand. The nanoparticles were then separated first by applying a strong magnet and subsequently by centrifugation to collect the precipitate. This washing/separation cycle was repeated at least three times. The supernatant was removed, and the remaining sample was dried at 40 °C.

### 3.3. Nanoparticle Activation and HRP Immobilization

EDAC activates carboxyl groups of HRP, which subsequently react with the amino groups of surface-bound 5ASA to form covalent amid linkages. The nanomaterials were treated with 22 mg of EDAC in 18 mL of Tris-HCl buffer (50 mM, pH 8.5) for 30 min at 25 °C under gentle stirring (150 rpm) to activate Fe_3_O_4_ nanomaterials, following the optimized procedure from our previous work [22]. These parameters, including EDAC concentration, activation time, and pH, were selected to ensure a strong covalent attachment of the enzyme and high retention of activity. The chosen pH of 8.5 provides a balance between maintaining HRP structural stability and ensuring deprotonation of the amino groups of 5ASA (pKa ≈ 5.8), which promotes nucleophilic attack on the enzyme’s carboxyl groups and enables amide bond formation. Afterward, 10, 25, 50, 75, or 100 μL (depending on whether the amount of particles or enzyme is varied) of HRP solution was added to a vessel containing 10, 15, 20, and 30 mg of activated nanomaterials, along with 990, 975, 950, 925 or 900 μL of Tris-HCl buffer (50 mM, pH 8.5), 1 mL od EDAC. The final volume of the enzyme-support suspension was fixed at 2.0 mL for all immobilization experiments. Immobilization was carried out in 2 mL polypropylene microcentrifuge tubes (Eppendorf), which allowed efficient mixing under agitation and straightforward magnetic separation of the nanomaterials. The initial protein loading was adjusted by varying the volume and concentration of the HRP solution, resulting in enzyme-to-support ratios ranging from approximately 80 to 1250 mg of HRP per gram of nanomaterial, depending on the experimental conditions. Immobilization was performed for 20 h at 25 °C under gentle magnetic stirring (150–200 rpm, depending on the number of samples processed simultaneously) to ensure homogeneous nanoparticle dispersion and uniform enzyme-support contact. The main stages of the immobilization procedure are schematically illustrated in Figure S1e (Supplementary Information). Control experiments performed with bare Fe_3_O_4_ nanoparticles under identical conditions showed no detectable catalytic activity.

As a control, free HRP was incubated under identical immobilization conditions (temperature, time, agitation, and buffer composition) in the absence of nanoparticles. No significant loss of enzymatic activity was detected after 20 h, indicating that the immobilization conditions themselves do not induce measurable enzyme inactivation.

### 3.4. Determination of Protein Concentration Using the Bradford Method

Immobilization efficiency (mg g^−1^) was calculated as the difference between the initial amount of enzyme added to the reaction mixture and the amount remaining in the supernatant after immobilization, divided by the nanomaterial’s dry mass (g). It should be noted that this method provides an estimation of immobilization efficiency based on the post-immobilization supernatant and may not fully account for minor protein losses during handling; however, it is suitable for comparative analysis between immobilization systems. The amount of enzyme in both the initial and final solutions was determined using the Bradford assay. Protein concentration was determined using the Bradford assay reagent, prepared from Coomassie-Brilliant Blue G-250 according to standard protocols [22]. Briefly, 40 μL of the sample was mixed with 2 mL of diluted dye reagent, and absorbance was measured after incubation. The dye reagent was prepared in-house by diluting a concentrated Coomassie Brilliant Blue G-250 solution according to the established procedure. The enzyme concentration in solution was determined both before and after immobilization using the described method. The difference in enzyme concentration was then calculated on a per-gram basis for the nanomaterial.

### 3.5. Determination of Enzyme Activity

The enzymatic activity of immobilized horseradish peroxidase (HRP) was assessed using pyrogallol as the standard substrate in the presence of hydrogen peroxide (H_2_O_2_). The reaction was monitored spectrophotometrically at 420 nm, corresponding to the formation of purpurogallin, the oxidative product of pyrogallol. The assay was conducted using a UV-Vis spectrophotometer (UV-1700, Shimadzu Corporation, Kyoto, Japan), which detected the color change from yellow (pyrogallol) to dark violet-brown (purpurogallin).

The reaction mixture consisted of 13 mM pyrogallol in 0.1 M phosphate buffer (pH 7.0), 3% (*v*/*v*) hydrogen peroxide, and varying quantities (10, 15, 20, or 30 mg) of HRP-immobilized magnetic nanoparticles. A blank control was prepared using the same pyrogallol solution in phosphate buffer without adding hydrogen peroxide. The absorbance of the reaction mixture was recorded every 15 s over 3 min, with measurements taken relative to the blank.

The total enzyme activity expressed in U is determined based on the following expression:

(1) $$A_{\mathrm{IE}}\;=\;\frac{\Delta A\times V_{t}}{\Delta t\times \varepsilon }$$

$\frac{\Delta A}{\Delta t}$—absorbance change per minute,

ε—the molar extinction coefficient of pyrogallol at 420 nm (12 L mmol^−1^)

*V_t_*—total volume of the reaction mixture (mL)

The specific activity of the immobilized enzyme (Ug^−1^) was calculated relative to the mass of the nanoparticle carrier. One unit (1 U) of peroxidase activity was defined as the amount of enzyme that catalyzes the oxidation of 1 µmol of pyrogallol to purpurogallin per minute under the assay conditions (25 °C, pH 7.0). The activity was calculated from the change in absorbance at 420 nm using the molar extinction coefficient of purpurogallin (ε = 12 L mmol^−1^ cm^−1^). Representative photographs of the experimental setup and assay are provided in Figure S1f–h (Supplementary Information). Reusability was assessed by magnetically separating the nanobiocatalyst after each cycle, washing it with deionized water, and reusing it under identical assay conditions for up to five consecutive runs.

All experiments were performed in triplicate, and results are expressed as mean ± standard deviation (SD).

### 3.6. Characterization of Fe_3_O_4_ Nanomaterials

The XRD pattern of Fe_3_O_4_ nanoparticles was recorded using a Rigaku SmartLab (Tokyo, Japan) diffractometer (Cu-Ka_1,2_ radiation, λ = 0.1540 nm) in a 2θ range of 10 to 90° with a 0.02° step and 1° min^−1^ counting time. Fourier transform infrared (FTIR) spectroscopy measurements of the Fe_3_O_4_, Fe_3_O_4_/5ASA, and Fe_3_O_4_/CA hybrid nanomaterials were performed before and after enzyme immobilization using a Thermo Nicolet (Waltham, MA, USA) 6700 FTIR spectrometer.

High-resolution transmission electron microscopy (HRTEM) was used to investigate the morphology and fine structural characteristics of the synthesized materials. Analyses were conducted using an instrument operating at 300 kV with a LaB_6_ cathode as the electron source, enabling a theoretical resolution of 1.7 Å. The system was equipped with a GATAN MULTISCAN (Pleasanton, CA, USA) CCD camera (model 794) and an energy-dispersive X-ray spectroscopy (EDS) module (INCA x-stream, Oxford Instruments, Abingdon, UK). For sample preparation, the powders were ultrasonically dispersed in ethanol, and a small volume of the resulting dilute suspension was deposited onto a copper grid coated with holey carbon, followed by air-drying at room temperature.

Magnetic measurements were performed using an MPMS XL-5 SQUID magnetometer (Quantum Design, San Diego, CA, USA). Isothermal magnetic curves were measured at 300 K in the −50 kOe to 50 kOe magnetic field range.

## 4. Conclusions

In this study, horseradish peroxidase (HRP) was successfully immobilized onto Fe_3_O_4_ nanoparticles functionalized with 5-aminosalicylic acid (5ASA) and caffeic acid (CA), using a simple and efficient covalent coupling strategy. The Fe_3_O_4_/5ASA system enabled covalent binding of the enzyme, resulting in significantly higher immobilization efficiency, catalytic activity (>100 U/g), and operational stability compared to the non-covalently bound Fe_3_O_4_/CA system. The Fe_3_O_4_/5ASA nanobiocatalysts maintained competitive activity after five reuse cycles, with residual activity still surpassing that of the initial Fe_3_O_4_/CA system and many non-covalent immobilization platforms reported in the literature. This highlights their robustness, operational stability, and suitability for applications requiring reusable biocatalysts. In addition to excellent stability and reusability, the magnetic properties of the Fe_3_O_4_-based carrier allow for rapid and efficient separation from reaction mixtures using an external magnetic field, making this system highly practical for repeated or continuous biocatalytic applications. These results confirm the crucial role of surface functionalization in preserving enzyme structure and function, and highlight the potential of 5ASA-modified Fe_3_O_4_ nanoparticles as a robust, magnetically recoverable, and reusable platform for enzyme immobilization in various biotechnological applications.

## Data Availability

The data presented in this study are available on request from the corresponding author.

## Supplementary Materials

The following supporting information can be downloaded at: https://www.mdpi.com/article/10.3390/molecules31010178/s1, Figure S1: Pictures of every step of the synthesis of Fe_3_O_4_ nanoparticles; Table S1: Amount of immobilized HRP enzyme.
