# Supplementary material for: Magnetically Recoverable ICT-Functionalized Fe3O4 Nanoparticles for Efficient Horseradish Peroxidase Immobilization
Source: Molecules. 2026 Jan 2;31(1):178. doi: 10.3390/molecules31010178 (PMC12787986; doi:10.3390/molecules31010178)
Supplement: Supplementary file 1 [file molecules-31-00178-s001.zip › molecules-4050478-supplementary.pdf]

Supporting Information

Magnetically Recoverable ICT-Functionalized Fe_3O_4 Nanoparticles for Efficient Horseradish Peroxidase Immobilization

Katarina Isaković [1], Marko Jonović [2], Dušan Sredojević [1,*], Marko Bošković [3], Jovana Periša [1], Zorica Knežević-Jugović [4] and Vesna Lazić [1,*]

1 Vinča Institute of Nuclear Sciences, National Institute of the Republic of Serbia, Centre of Excellence for Photoconversion, University of Belgrade, 11001 Belgrade, Serbia; katarina.isakovic@vin.bg.ac.rs (K.I.); jburojevic@yahoo.com (J.P.)
2 Institute of Chemistry, Technology and Metallurgy, University of Belgrade, 11000 Belgrade, Serbia; marko.jonovic@ihtm.bg.ac.rs
3 Laboratory for Theoretical and Condensed Matter Physics, Vinča Institute of Nuclear Sciences, National Institute of the Republic of Serbia, University of Belgrade, 11001 Belgrade, Serbia; markob@vin.bg.ac.rs
4 Department of Biochemical Engineering and Biotechnology, Faculty of Technology and Metallurgy, University of Belgrade, 11000 Belgrade, Serbia; zknez@tmf.bg.ac.rs
* Correspondence: dusanmcrae@yahoo.com (D.S.); vesna.lazic@vin.bg.ac.rs (V.L.)

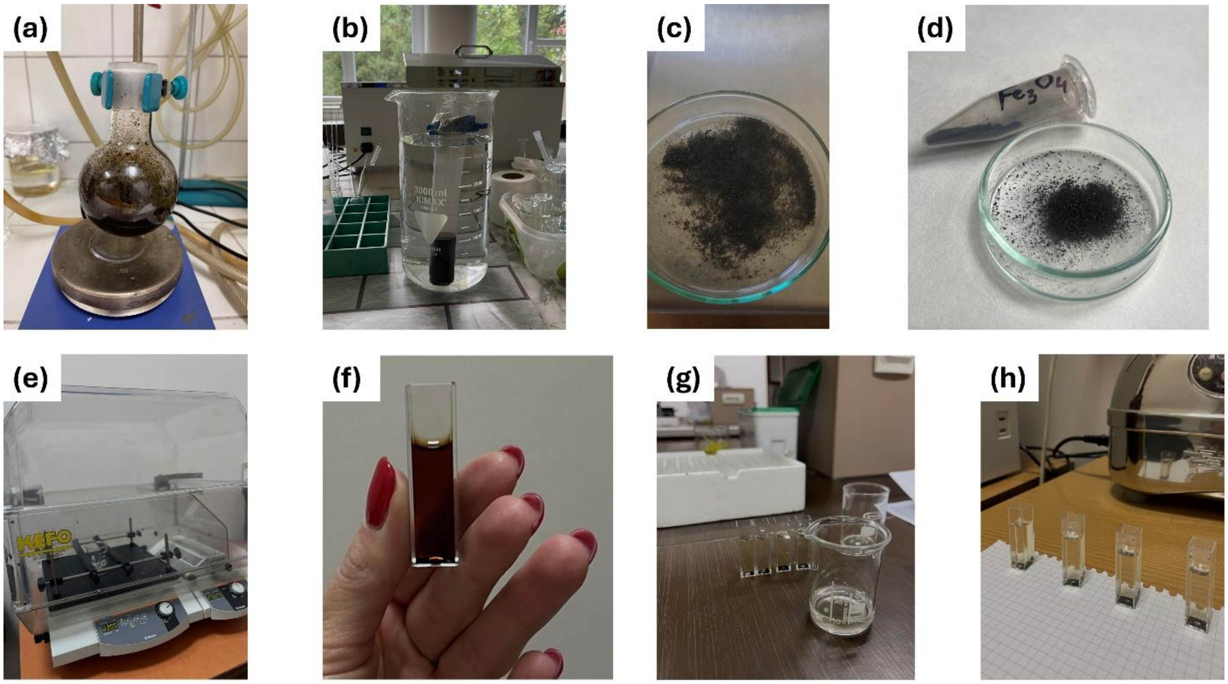

Figure S1. Pictures of every step of the synthesis of Fe_3O_4 nanoparticles (a-d), immobilization of HRP (e), and the experiment of activity (f-h).

Table S1. Amount of immobilized HRP enzyme onto Fe_3O_4/5ASA and Fe_3O_4/CA nanomaterials

Sample	Concentration of HRP enzyme (μg mL^{-1})	Amount of nanomaterials (mg)	Amount of immobilized HRP enzyme per 1 g of nanomaterials (mg g^{-1})	Amount of HRP enzyme in the reaction mixture (mg)
Fe_3O_4/5ASA/HRP	250	10	243.4±3.3	2.43±0.03
		15	380.9±6.6	5.71±0.10
		20	1068.7±2.9	21.4±0.06
		30	888.8±4.4	26.7±0.13
	25	10	439.2±0.7	4.39±0.01
	62.5		433.9±1.2	4.34±0.01
	125		418.0±1.0	4.18±0.01
	187.5		439.2±1.6	4.39±0.01
Fe_3O_4/CA/HRP	250	10	0.26±0.05	0.003±0
		15	≤ 0.10	ND
		20	≤ 0.10	ND
		30	≤ 0.10	ND
	25	10	2.85±0.04	0.028±0
	62.5		2.43±0.03	0.024±0
	125		1.27±0.02	0.013±0
	187.5		≤ 0.10	ND

*ND – not detected; below the detection limit of the Bradford assay (≤ 0.10 mg/g).
